# Variability of the Sprint Step Movement Pattern and Its Association with Hamstring Injury Risk

**DOI:** 10.3390/jcm15010281

**Published:** 2025-12-30

**Authors:** Mateusz Jopek, Michal Krzysztofik, Dariusz Mroczek, Adam Zajac, Krzysztof Mackala

**Affiliations:** 1 Department of Individual and Team Physical Activities, Wroclaw University of Health and Sport Sciences, al. Ignacego Jana Paderewskiego 35, 51-612 Wrocław, Poland; mateusz.jopek@awf.wroc.pl; 2 Institute of Sport Sciences, The Jerzy Kukuczka Academy of Physical Education in Katowice, Mikołowska 72A, 40-065 Katowice, Polanda.zajac@awf.katowice.pl (A.Z.); 3 Department of Sport Games, Faculty of Physical Education and Sport, Charles University, 162 52 Prague, Czech Republic; 4 Department of Biological and Motor Bases of Sports, Wroclaw University of Health and Sport Sciences, al. Ignacego Jana Paderewskiego 35, 51-612 Wrocław, Poland; dariusz.mroczek@awf.wroc.pl

**Keywords:** biomechanics, elite athletes, hamstring injury, injury prevention, kinematic variability, sprinting

## Abstract

**Background:** This study investigated how kinematic parameters vary during repeated 50 m sprints and their relationship with movement stability and hamstring injury risk among sprinters at different competitive levels. **Methods**: Eighteen male Polish National Team sprinters (nine elite, nine sub-elite) performed four 50 m sprints, with measurements of step length, frequency, ground contact time, and flight time taken using the OptoJumpNEXT system across the entire track. The fastest and slowest trials were analyzed, and a 26-question survey examined participants’ hamstring injury history and prevention strategies. **Results:** Results showed that elite sprinters posted faster times, higher step frequency, shorter ground contact times, and increased step velocity, indicating more stable, consistent sprint mechanics. About 89% of athletes reported previous hamstring injuries, mainly during the late swing phase or between 40 and 50 m. The highest injury rate occurred during the fourth repetition, highlighting fatigue as a key risk factor. Elite sprinters mainly increased speed through higher step frequency and shorter ground contact times, while sub-elite athletes relied more on longer step lengths. **Conclusions**: Overall, elite sprinters exhibit more stable and efficient movement patterns, which may reduce the risk of hamstring strain injury. In contrast, the greater variability and fatigue seen in sub-elite athletes could heighten their injury vulnerability.

## 1. Introduction

Research highlights running technique as a critical factor in sprinters’ performance [1,2]. During sprint speed training, kinematic parameters vary due to fatigue, muscular adaptation, altered force application, shorter step lengths, and changes in body lean angle [1,3]. As sprint repetitions and intensity increase, these changes can compromise the stability of movement patterns, which is defined as the ability to maintain optimal motion despite varying conditions. Stability is a key determinant of both performance and injury risk [4,5,6]. Higher-level sprinters tend to exhibit less kinematic variability during prolonged efforts, indicating greater movement stability [1,3]. Understanding these variations is essential for optimizing training, enhancing performance, and minimizing injury risk [6,7,8].

Unstable movement patterns can increase the risk of injury. Deficits in sprinting technique, especially during late swing or early stance phases, are closely linked to hamstring strain injuries [9,10]. Alterations in running form, such as excessive hip flexion or overlong steps, can raise muscle tension and injury chances [11,12,13]. Stretch injuries, caused by excessive muscle elongation under active tension, are common, particularly when significant hip flexion occurs with knee extension. The late swing phase of sprinting places the most stress on the hamstring muscles [14]. During sprint acceleration, the hamstrings undergo greater and more rapid lengthening than at a steady pace, increasing injury risk [8,15]. Fatigue-related changes in movement and reduced muscle activity can impair lower-limb control during the final running phase, increasing the likelihood of injury [6,7,8].

The hamstrings, particularly the long head of the biceps femoris (BFlh), are critical during sprinting’s acceleration and deceleration phases and are highly susceptible to injury due to their combined eccentric and concentric actions. During acceleration, the hamstrings experience faster stretch velocities and greater lengthening than in constant-speed running, further stressing the muscle-tendon complex [8]. Modeling and electromyographic studies show that the BFlh endures extreme tensile loads during simultaneous hip flexion and knee extension [12,16]. In summary, the hamstrings face intense mechanical demands during sprinting, with peak overload occurring in the late swing phase, when the muscle is maximally stretched and eccentrically active, increasing the risk of microtrauma or tears [17,18].

Recent studies show that intense sprint sessions can temporarily weaken posterior chain muscles and cause increased anterior pelvic tilt. This may elevate hamstring strain and injury risk [19]. This article examines how kinematic parameters vary between the fastest and slowest sprints, reflecting intra-session performance changes. This method reveals variability across multiple sprints and explores its possible impact on movement stability and hamstring injury risk among sprinters at various competitive levels.

## 2. Materials and Methods

### 2.1. Participants

The study carefully selected 18 male sprinters, evenly split between elite (seniors with a 100 m personal best of ≤10.40 s) and sub-elite (juniors with a 100 m personal best of ≤11.10 s) groups. All participants were valued members of the Polish National Track and Field Team, ensuring high expertise and experience. The elite group (n = 9) had a mean age of 23.1 ± 3.7 years, a height of 1.82 ± 0.1 m, a body weight of 76.7 ± 8.0 kg, and a BMI of 23.2 ± 1.1 kg/m^2^. Their average personal bests were 6.67 ± 0.1 s for 60 m and 10.29 ± 0.1 s for 100 m. The sub-elite group (n = 9) was notably younger (18.4 ± 0.9 years) and lighter (67.6 ± 6.9 kg, BMI = 21.2 ± 1.2 kg/m^2^), with mean best times of 7.05 ± 0.2 s for 60 m and 10.75 ± 0.2 s for 100 m. All participants gave written informed consent, with parental consent obtained for minors. The study was approved by the Bioethics Committee for Scientific Research at the Jerzy Kukuczka Academy of Physical Education in Katowice (Approval No. 3/17 June 2021). It was conducted in accordance with the Declaration of Helsinki (2013). The project received funding from the Polish Ministry of Sport and Tourism, supporting the study’s credibility.

### 2.2. Study Procedure

Testing was conducted in January 2023 during a national sprint team training camp under ideal physical and environmental conditions. All evaluations took place indoors at the Central Olympic Preparation Center in Spała. Each sprinter performed four 50 m sprints under standardized recovery conditions. For comparison, the fastest and slowest trials were selected to showcase the range of intra-session performance, instead of analyzing every repetition. This method helped evaluate the variability between peak and fatigued performances. Sprint times and kinematic data were recorded using the OptoJumpNEXT system (Microgate, Bolzano, Italy), and total sprint time was measured with WittyGate photocells (Microgate). Participants also completed a questionnaire on hamstring injury history over the past 3–5 years to aid biomechanical analysis. Anthropometric measurements, including height, body mass, and BMI, were taken at the start of testing.

### 2.3. Hamstring Injury Survey

The survey consisted of 26 predominantly closed-ended questions (multiple-choice and scale items) with conditional and subjective elements. Questions 1–2 included general information and consent confirmation, while the substantive sections were logically divided into three thematic parts: demographics, injury history, and training/prevention.

Introductory section (Questions 1–2): Confirmation of informed consent and competitive experience level.

Demographics (Questions 3–7): Closed-ended questions that identify age, training background, sport type, and competition level.

Injury History (Questions 8–17): Conditional and categorical questions about the occurrence, side, severity, and sprint segment of hamstring injuries.

Training and Prevention (Questions 18–26): Mixed-format questions about sprint training structure, repetition counts (2–6), prevention routines, and how often muscle tightness episodes occur.

This structure provided thorough coverage of both descriptive and perceptual data while ensuring a logical flow across survey sections. Internal reliability (Cronbach’s α = 0.78) was computed only for the six items in the “Training and Prevention” section, which assessed perceptions of injury risk, prevention effectiveness, and muscle tightness frequency. These items formed a coherent Likert-type scale reflecting athletes’ subjective views on preventive practices. The other categorical questions were not included in this analysis. To ensure all participants understood the technical terms used in the survey (e.g., late swing, early stance, or specific sprint segments), the questionnaire included brief explanations and schematic diagrams illustrating the relevant phases of the sprint cycle. The survey was administered under the supervision of the researcher and team coach, who clarified any terms as needed. This approach ensured a consistent understanding of technical terminology among respondents.

### 2.4. Kinematic Analysis of the 50 m Sprint

Performance data were collected using the OptoJump NEXT system (Microgate, Bolzano, Italy), which has parallel LED bars along the 50 m track to record each ground contact and flight phase with millisecond accuracy. Sprint times were also measured separately with WittyGate photocells (Microgate, Bolzano, Italy) at 0 m, 10 m, 20 m, 30 m, and 50 m to determine split times and total duration. The OptoJump tracked step length, step frequency, ground contact time, and flight time across the entire 50 m, documenting each step for each athlete. The number of steps generally ranged from 24 to 26, depending on speed and technique, with measurements at 1 ms precision (sampling rate: 1000 Hz). Its LED grid (1 cm resolution) enabled accurate, contactless detection of foot strikes. Sprinters started each run from a semi-crouched position to promote quick, smooth acceleration. A standardized warm-up included two 40 m runs at 85–90% effort. Each athlete performed four runs, with five-minute breaks between. The fastest and slowest runs were analyzed to compare stride kinematics across speeds, providing a detailed view of sprint biomechanics.

### 2.5. Statistical Analysis

Data were analyzed using Statistica software 14.1.0 (StatSoft, Inc., Tulsa, OK, USA). Normality was tested with the Shapiro–Wilk test. Variance homogeneity was assessed using Levene’s test. Group comparisons employed Student’s *t*-test for independent samples. To mitigate potential inflation of type I error from multiple comparisons across numerous kinematic variables, the false discovery rate (FDR) approach via the Benjamini–Hochberg procedure was used to adjust *p*-values where appropriate. This method provides a balanced control of error rates without being as conservative as the Bonferroni correction. A two-way mixed-model ANOVA (repeated measures) examined the effects of two factors on sprinters’ kinematic performance: competitive level (between-subject: elite vs. sub-elite) and run type (within-subject: slow vs. fast trial, repeated within each athlete). This model accounts for the dependence of observations from each athlete performing both sprint conditions. When significant main effects or interactions emerged, Tukey’s HSD post hoc test was applied for pairwise comparisons. Levene’s test verified homogeneity of variances, and the Shapiro–Wilk test assessed normality. Pearson’s correlation coefficients explored linear relationships between continuous variables, with results displayed as correlation matrices. Survey responses were summarized with descriptive statistics, including frequencies and percentages, to illustrate injury prevalence, prevention practices, and subjective perceptions among athletes. No formal a priori sample size calculation was conducted, as the study included all available members of the national and sub-elite sprint teams meeting the inclusion criteria. The small sample size reflects the limited population of high-performance sprinters and should be considered when generalizing the results.

## 3. Results

### 3.1. Survey—Analysis of Key Factors

**Figure 1:**
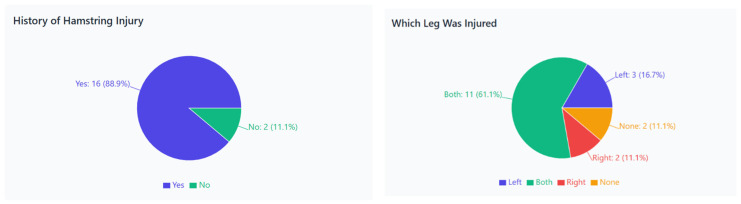
Prevalence and laterality of hamstring injuries among sprinters. (**Left**: history of hamstring injury—percentage of athletes reporting previous injuries. **Right**: injured leg distribution—bilateral, left, or right side involvement).

The survey revealed a high prevalence of biceps femoris injuries during sprint training, particularly in sessions designed to develop maximum speed. This finding underscores the significant risk of overload inherent to sprint training, which can lead to these injuries. A total of 89% of athletes reported having a previous hamstring injury, with 61% experiencing bilateral injuries. Unilateral injuries were less common, affecting the left limb in 17% and the right limb in 11% of respondents. These results confirm a substantial risk of overload inherent to sprint training (Figure 1).

**Figure 2:**
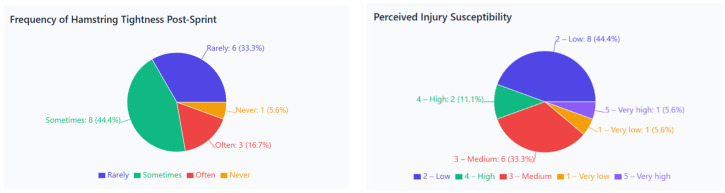
Frequency of post-sprint hamstring tightness and perceived susceptibility to injury among sprinters. (**Left**: frequency of hamstring tightness episodes following high-intensity sprints. **Right**: athletes’ self-assessed risk level of sustaining a hamstring injury).

After high-intensity sprints, 77% of athletes reported experiencing episodes of hamstring tightness or a “pulling” sensation, while 23% said they had never felt such symptoms. Among those with symptoms, 44% experienced them “sometimes” and 33% “rarely,” whereas 6% of all respondents reported “never” experiencing such sensations. These results suggest that minor tightness after sprints is common and may be an early warning sign of possible muscle overload (Figure 2).

**Figure 3:**
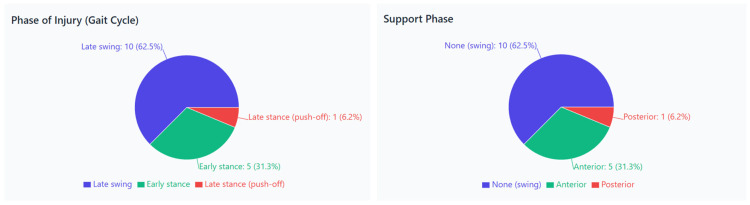
Phases of sprinting associated with hamstring injury occurrence. (**Left**: distribution of injuries across gait cycle phases—late swing, early stance, and late stance. **Right**: distribution of injuries by support phase—none [swing], anterior, and posterior support.

Figure 3 shows that most injuries occurred during the late swing phase (62.5%), followed by the early stance phase (31.3%) and the late stance phase (push-off) (6.2%). The correct chart indicates that 62.5% of injuries occurred during the swing phase, with 31.3% in the anterior support phase and 6.2% in the posterior support phase.

**Figure 4:**
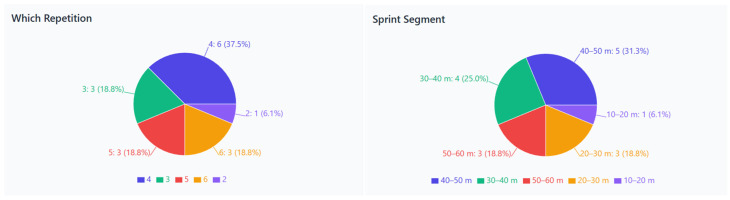
Distribution of hamstring injuries across sprint repetitions and sprint distance segments. (**Left**: frequency of hamstring injuries by sprint repetition number. **Right**: occurrence of injuries across sprint distance segments from 10 m to 60 m).

The injury data specific to repetitions and distances (2–6 repetitions; 10–60 m segments) were collected from athletes’ survey responses about their typical sprint-training experiences, not from the controlled experimental protocol. Figure 4 indicates that the 40–50 m segment had the highest injury occurrence (31.3%), followed by the 20–30 m and 30–40 m segments (each 18.8%), the 50–60 m segment (18.8%), and the 10–20 m segment (6.1%). It also shows that, based on survey responses, the fourth repetition during regular sprint training was the most common time for injury (37.5%), with the third, fifth, and sixth repetitions each at 18.8%, and the second at 6.1%. The questionnaire formulated response options starting from the second repetition because the first sprint was viewed as a warm-up or preparatory run, not a typical injury situation. These data reflect athletes’ retrospective self-reports during their standard training sessions, not the experimental protocol, which involved four 50 m sprints. This clarification indicates that the injury frequency data related to repetitions highlight perceived risk during regular sprint practice rather than outcomes from the kinematic testing session. Overall, these findings indicate that hamstring injuries are most common during later repetitions of maximum effort sprints, emphasizing fatigue and eccentric loading as key contributing factors.

### 3.2. Kinematic Analysis

Significant performance differences were found between elite and sub-elite sprinters (Table 1). Elite athletes recorded an average 50 m time of 5.427 ± 0.10 s, compared to 5.627 ± 0.13 s among sub-elite athletes (*p* = 0.0019). Although step count and step length differences were not statistically significant (*p* > 0.05), elite sprinters demonstrated higher step frequency in fast runs (*p* = 0.0080), shorter ground contact times (*p* = 0.0204), and greater step velocities (*p* < 0.0003), indicating more effective sprint mechanics and greater running speed consistency.

A two-way mixed-model ANOVA with repeated measures (competitive level × run type) was conducted, considering “run type” as a within-subject factor since each athlete performed both sprint conditions (Table 2). The competitive level had a significant impact on several variables, with elite sprinters exhibiting shorter run and step times and higher velocities. Specifically: time_50 (run time): significant effects of competitive level (*p* < 0.001) and run type (*p* = 0.048); single_st_time_50 (single step time): considerable effect of competitive level (*p* < 0.001); ground contact time: shorter among elites (*p* < 0.001); step velocity: higher among elites (*p* < 0.001). No significant interaction effects were found between competitive level and run type (all *p* > 0.8), indicating that differences between elite and sub-elite groups were consistent across both run types.

**Figure 5:**
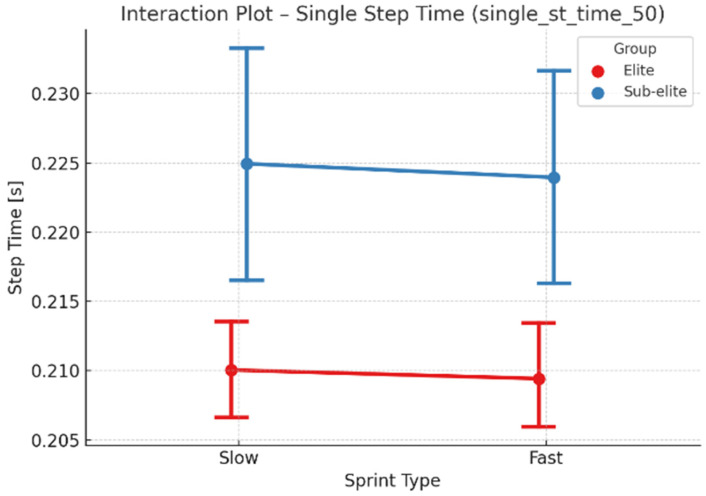
Interaction Plot—Single Step Time (single_st_time_50) for Elite and Sub-Elite Groups Across Slow and Fast 50 m Sprints.

The interaction plot for single-step time (Figure 5) showed parallel trends between groups, confirming the absence of interaction. This finding suggests that the design of training programs can be consistent across both elite and sub-elite groups, as the differences in movement mechanics are consistent across both run types. Elite sprinters achieved shorter step times in both slow and fast runs, reflecting more economical and efficient movement mechanics.

Tukey’s post hoc test for single_st_time_50 confirmed significant differences (*p* < 0.05) between most pairwise comparisons (Table 3). Elite sprinters consistently showed shorter step times than sub-elite athletes, both within and across run types (e.g., Elite_Fast vs. Sub-elite_Fast, Elite_Slow vs. Sub-elite_Fast). These findings reinforce the superior movement efficiency of elite performers, highlighting the importance of biomechanical analysis in sprint training.

**Figure 6:**
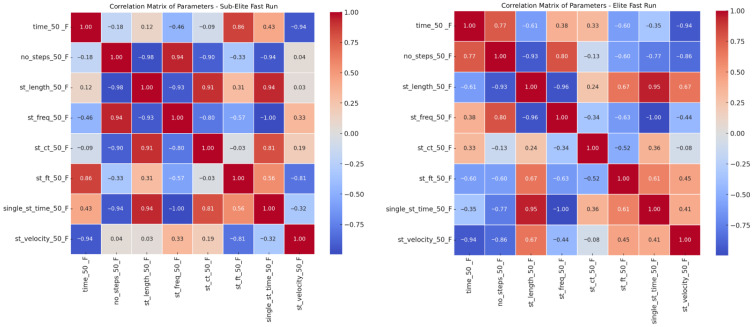
Correlation matrices of kinematic parameters during the 50 m fast sprint for Sub-Elite (**left**) and Elite (**right**) sprinters.

In the elite group, strong positive correlations (Figure 6) were found between step count and step frequency (r = 0.80), and moderate negative correlations between run time and step length/velocity (r = −0.48 to −0.35). This suggests that faster elite sprinters achieve higher speeds through a combination of step frequency and optimal step length. In contrast, the sub-elite group exhibited strong positive correlations between step length and single-step time (r = 0.94) and between step length and run velocity (r = 0.91), along with a moderate negative correlation between step frequency and velocity (r = −0.81). Thus, sub-elite athletes rely more on longer steps than on increased frequency to achieve higher speed.

## 4. Discussion

Although the experimental design included four sprint repetitions, the kinematic analysis concentrated on the fastest and slowest trials to capture intra-session variability in movement patterns rather than fatigue-related changes. This strategy offers a representative comparison between the most and least efficient motor patterns within the same session and across different competitive levels (elite and sub-elite), enabling an evaluation of the stability and consistency of sprinting technique under various performance conditions. The findings provide valuable insights into sprinting biomechanics and injury prevention. Notably, observable differences in kinematics between elite and sub-elite sprinters affect both performance and injury risk. To examine the link between movement patterns and biceps femoris injuries, sprinters completed a survey on maximum-speed running. Due to limited access to detailed medical records, this survey investigated biceps femoris injuries during high-speed running. While some subjective bias may remain, the six-item “Training and Prevention” subscale showed acceptable internal reliability (Cronbach’s α = 0.78), indicating consistent responses regarding perceived injury risk and preventive measures. The survey also collected key data on the occurrence and types of hamstring tendon injuries in sprinters.

Results indicated that 89% of respondents had experienced hamstring strain injuries, with 61% being bilateral, highlighting the hamstring muscles’ vulnerability to overload during high-speed sprint training. These findings are crucial for understanding the problem. The next step is to identify the underlying causes. While multiple factors may contribute, our analysis focused on the two or three most significant ones. Data shows injuries can occur early or late in training sessions, with the highest rate after the fourth repetition (37.5%), followed by the third, fifth, and sixth repetitions at 18.8% each. Injuries often happened during the final phase of a 40–50 m sprint, which was the most common injury site (31.3%). Two key factors likely involved are the quality of warm-up before training and the level of fatigue and training load associated with it.

Numerous studies [20,21] indicate that inadequate warm-ups increase the risk of hamstring injuries by reducing muscle elasticity. This decrease hampers force absorption and raises the likelihood of muscle damage, especially during high-speed movements. Such insufficient preparation can make muscles more susceptible to strain, particularly in the early stages of practice or competition [22]. It is often linked to other risk factors, such as muscle weakness and poor flexibility. In the current sample, these factors are unlikely to be primary causes among elite sprinters, but they may play a somewhat larger role in less experienced sub-elite athletes. This interpretation should be considered exploratory, as the current data were not divided to confirm these subgroup differences statistically.

Fatigue increases the risk of hamstring injuries in sprinters by decreasing muscle strength, reducing activation, and increasing muscle strain [23,24]. These neuromuscular changes caused by fatigue make the hamstrings more vulnerable during high-intensity, late-phase sprinting. Therefore, selecting the appropriate volume of speed training per session is essential [6,9]. This volume is often customized based on the athlete’s performance level and training cycle stage. Currently, there is no precise tool to determine the optimal sprint-training volume. Still, previous reports suggest that a typical high-intensity sprint session covers between 260 and 300 m of total sprinting distance, depending on performance level and fatigue tolerance [6,25,26]. Such training loads are commonly used to maintain high sprint quality while limiting excessive neuromuscular fatigue. Young athletes, like our juniors, are particularly at risk. Overloading can alter sprinting mechanics and increase injury risk [7]. Some ongoing (yet unpublished) experiments measure peak power after each repetition, which could help identify when to stop training. A noticeable drop in peak power may indicate the need to end the session, but no standard for a significant decline has yet been established.

Fatigue greatly reduces the hamstring muscles’ ability to produce force and activate effectively during intense sprints. Our research also revealed that 62.5% of injuries occur during the swing phase, consistent with previous studies showing that this is when the hamstrings are most at risk due to eccentric loading and rapid stretching [8,27,28,29]. Nagahara et al. [25] noted that differences in sprinting mechanics among athletes can affect force distribution across the hamstrings, thereby increasing injury risk. Microtears often occur during the late swing phase when the muscle is heavily stretched [8,30]. Moreover, injuries on the left leg were more frequent (17%) than on the right (11%), possibly reflecting asymmetries in running form or unilateral overload [12,18]. Consequently, fatigue from sprinting or training, combined with the high-intensity demands of sprinting, can alter sprint kinematics and increase the risk of hamstring injuries. 

Altered sprinting kinematics involve modifications in core movement parameters that improve the running motor pattern. This includes repeatedly performing high-intensity running steps. Better motor patterns lead to improved performance and a lower injury risk. Kinematic analysis shows differences between elite and sub-elite sprinters. Elite sprinters averaged a 50 m time of 5.427 ± 0.10 s, compared to 5.627 ± 0.13 s for sub-elites (*p* = 0.0019), demonstrating a clear advantage. These time differences indicate superior running technique among elites, as research links faster sprints to more efficient mechanics [31,32]. Elite sprinters also had shorter ground contact times (*p* = 0.0204) and higher step speeds (*p* < 0.0003), reflecting greater technical efficiency. Mendiguchia et al. [26] observed that elite sprinters exhibit more consistent kinematic patterns across repeated sprints, which aids injury prevention. Regarding stride frequency, elites showed higher rates, especially at faster speeds (*p* = 0.0080). This supports findings that top sprinters accelerate by taking longer strides and increasing cadence, indicating a more dynamic and refined running technique [7,17].

A key finding of this study is the significant difference in single-stride time between elite and sub-elite groups. This likely affects the support phase, during which hamstring problems often occur [33]. This factor further distinguishes sprinters’ performance levels. Elite sprinters exhibit shorter stride times at both slow and fast paces, indicating more efficient running mechanics. A reduced stride time signals better technical efficiency, enabling sprinters to cover more distance in less time [1,3]. Musculoskeletal simulations demonstrate how lower-limb muscles coordinate to maximize propulsive efficiency during accelerated sprinting [34]. Although this model focuses on performance optimization rather than injury mechanisms, it provides valuable biomechanical context for understanding the mechanical demands on the hamstrings during high-speed running. Step speed analysis reveals that elite sprinters average 9.208 m/s, compared to 8.874 m/s for sub-elite sprinters, suggesting that elite athletes generate greater horizontal force, thereby enhancing sprinting performance [8,35]. These differences reflect that elite sprinters manage their mechanics and optimize force application better during sprinting, with superior trunk stabilization and proximal muscle activation [36]. Despite their improved running dynamics, high-level sprinters tend to experience fewer changes in muscle stiffness, activation, and function, making their hamstrings less susceptible to failure under high forces or strains. Recent EMG studies show that increasing running speed alters the within-muscle excitation distribution in the biceps femoris, changing regional loading [37,38]. This supports the idea that ‘kinematic variability’—the extent of changes in stride length and ground contact time—is lower in elite sprinters. Conversely, sub-elite athletes display greater variability, indicating less stable running techniques. These findings align with previous research showing that higher-level athletes generally exhibit lower kinematic variability, which has been interpreted as a marker of greater movement control and coordination efficiency [12,39,40]. In this study, this pattern was observed in elite sprinters across both running types. However, it is essential to note that no direct statistical link was found between individual variability and injury history. Therefore, the idea that lower variability reduces injury risk remains hypothetical and is supported by existing literature rather than direct evidence. Future studies with prospective injury monitoring are needed to confirm whether the observed biomechanical consistency actually provides a protective benefit.

### Limitations and Future Directions

This study has some limitations to consider when interpreting the results. The relatively small number of participants reflects the limited availability of elite and sub-elite sprinters, which naturally restricts the generalizability of the findings. Data on hamstring injuries were collected retrospectively via self-reported questionnaires, potentially influenced by memory bias or personal interpretation. While the kinematic parameters were analyzed in detail, the study did not include physiological or neuromuscular measures, such as fatigue markers or electromyographic data, that could provide a more complete understanding of the mechanisms affecting sprint performance. Furthermore, the cross-sectional design prevents us from establishing causal relationships between movement variability and hamstring strain injuries.

Future research should focus on studying larger, more varied athletic groups, implementing prospective injury monitoring, and integrating biomechanical and physiological analyses to understand better how movement variability and fatigue influence hamstring injury risk in sprinting athletes.

## 5. Conclusions and Implications for Training

This study’s findings reveal significant kinematic differences between elite and sub-elite sprinters, linked to variations in performance and potentially different injury risk profiles. Elite sprinters showed improved technical efficiency, characterized by shorter ground contact times, higher step speeds, and less kinematic variability. While these features likely enhance performance, their potential role in protecting against hamstring injuries should be interpreted with caution, as the study’s cross-sectional design does not allow for causal inference.

When it comes to injury prevention, elite sprinters generally exhibit lower kinematic variability and more consistent running techniques, which likely help reduce the risk of acute hamstring strain injuries during maximal sprinting. This underscores the importance of refining running technique and neuromotor control in sub-elite sprinters to boost performance and reduce injury risk. Additionally, the study indicates that adding practical eccentric exercises, such as the Nordic hamstring exercise, to training routines can effectively prevent injuries and enhance performance.

## Figures and Tables

**Table 1:** Characteristics of kinematic parameters for the slowest and fastest 50 m sprint, divided into Elite and Sub-Elite, *p* < 0.05.

Variables		*n*	$\bar{x}$	s	v	Confidence		t	*p*
						−95%	+95%		
50 m Slow Run Time [s]	Elite	9	5.427	0.10	1.84	5.35	5.50	**−3.71**	**0.0019**
	Sub-elite	9	5.627	0.13	2.31	5.53	5.73		
50 m Fast Run Time [s]	Elite	9	5.351	0.11	2.06	5.27	5.44	**−3.49**	**0.0030**
	Sub-elite	9	5.544	0.12	2.16	5.45	5.64		
Number of Steps (Slow)	Elite	9	25.556	0.88	3.44	24.88	26.23	1.05	0.3100
	Sub-elite	9	24.889	1.69	6.79	23.59	26.19		
Number of Steps (Fast)	Elite	9	25.556	1.01	3.95	24.78	26.33	1.48	0.1572
	Sub-elite	9	24.778	1.20	4.84	23.85	25.70		
Step Length (Slow) [cm]	Elite	9	191.658	6.17	3.22	186.91	196.40	−1.30	0.2107
	Sub-elite	9	197.344	11.54	5.85	188.48	206.21		
Step Length (Fast) [cm]	Elite	9	192.243	7.16	3.72	186.74	197.75	−1.39	0.1831
	Sub-elite	9	198.144	10.51	5.30	190.06	206.23		
Step Frequency (Slow) [steps/s]	Elite	9	4.781	0.13	2.72	4.68	4.88	**2.93**	**0.0097**
	Sub-elite	9	4.472	0.29	6.48	4.25	4.69		
Step Frequency (Fast) [steps/s]	Elite	9	4.784	0.14	2.93	4.68	4.89	**3.03**	**0.0080**
	Sub-elite	9	4.484	0.26	5.80	4.28	4.69		
Contact Time (Slow) [s]	Elite	9	0.105	0.01	9.52	0.10	0.11	**−2.57**	**0.0204**
	Sub-elite	9	0.116	0.01	8.62	0.11	0.12		
Contact Time (Fast) [s]	Elite	9	0.104	0.01	9.62	0.10	0.11	**−2.72**	**0.0151**
	Sub-elite	9	0.115	0.01	8.70	0.11	0.12		
Flight Phase (Slow) [s]	Elite	9	0.105	0.01	9.52	0.10	0.11	−1.46	0.1642
	Sub-elite	9	0.109	0.01	9.17	0.10	0.12		
Flight Phase (Fast) [s]	Elite	9	0.105	0.01	9.52	0.10	0.11	−1.05	0.3079
	Sub-elite	9	0.109	0.01	9.17	0.10	0.11		
Single Step Time (Slow) [s]	Elite	9	0.210	0.01	4.76	0.21	0.21	**−2.90**	**0.0105**
	Sub-elite	9	0.225	0.01	4.44	0.21	0.24		
Single Step Time (Fast) [s]	Elite	9	0.209	0.01	4.78	0.20	0.21	**−3.07**	**0.0073**
	Sub-elite	9	0.224	0.01	4.46	0.21	0.23		
Step Velocity (Slow) [m/s]	Elite	9	9.183	0.14	1.84	9.08	9.29	**4.66**	**0.0003**
	Sub-elite	9	8.807	0.20	2.31	8.65	8.96		
Step Velocity (Fast) [m/s]	Elite	9	9.208	0.11	2.06	9.13	9.29	**4.75**	**0.0002**
	Sub-elite	9	8.874	0.18	2.16	8.73	9.01		

Bold values indicate statistically significant differences (*p* < 0.05).

**Table 2:** A two-way mixed-model ANOVA with repeated measures (competitive level × run type) of Kinematic Parameters for the Slowest and Fastest 50 m Run, Divided by Elite and Sub-Elite, *p* < 0.05.

Parameter	Group Effect (*p*)	Sprint Type Effect (*p*)	Interaction (*p*)
Running time	**0.0000**	**0.0482**	0.9191
Number of steps	0.0892	0.8936	0.8936
Step length	0.0658	0.8214	0.9721
Step frequency	0.0865	0.9868	0.8074
Ground contact time	**0.0007**	0.8127	0.9165
Flight phase	0.0865	0.9868	0.8074
Single-step time	**0.0002**	0.8190	0.9585
Step velocity	**0.0000**	0.3908	0.7021

Bold values indicate statistically significant differences (*p* < 0.05).

**Table 3:** Post Hoc Tukey’s Test Results for Mean Differences in Single Step Time (seconds) Between Elite and Sub-Elite Groups Across Fast and Slow 50 m Sprints.

Group 1	Group 2	Mean Difference	Lower CI	Upper CI	*p*-Value
Elite_Fast	Elite_Slow	0.0006	−0.0128	0.0140	0.9993
Elite_Fast	Sub-elite_Fast	0.0145	0.0011	0.0279	**0.0293**
Elite_Fast	Sub-elite_Slow	0.0155	0.0021	0.0289	**0.0180**
Elite_Slow	Sub-elite_Fast	0.0139	0.0005	0.0273	**0.0393**
Elite_Slow	Sub-elite_Slow	0.0149	0.0015	0.0283	**0.0245**

Bold values indicate statistically significant differences (*p* < 0.05).

## Data Availability

The data presented in this study are available on request from the corresponding author.

## References

[1] Morin J.B., Edouard P., Samozino P. (2011). Technical ability of force application as a determinant factor of sprint performance. Med. Sci. Sports Exerc..

[2] Weyand P.G., Sternlight D.B., Bellizzi M.J., Wright S. (2000). Faster top running speeds are achieved with greater ground forces, not more rapid leg movements. J. Appl. Physiol..

[3] Hunter J.P., Marshall R.N., McNair P.J. (2005). Relationships between ground reaction force impulse and kinematics of sprint-running acceleration. J. Biomech..

[4] Di Prampero P.E., Fusi S., Morin J.B., Belli A., Antonutto G. (2005). Sprint running: A new, energetic approach. J. Exp. Biol..

[5] Billaut F., Basset F., Falgairette G. (2005). Muscle coordination changes during intermittent cycling sprints. Neurosci. Lett..

[6] Romero V., Lahti J., Castaño Zambudio A., Mendiguchia J., Jiménez-Reyes P., Morin J.B. (2022). Effects of fatigue induced by repeated sprints on sprint biomechanics in football players: Should we look at the group or the individual?. Int. J. Environ. Res. Public Health.

[7] Bramah C., Mendiguchia J., Dos Santos T., Morin J.B. (2023). Exploring the role of sprint biomechanics in hamstring strain injuries: A current opinion on existing concepts and evidence. Sports Med..

[8] Bramah C., Rhodes S., Clarke-Cornwell A., Dos Santos T., Morin J.B., Mendiguchia J. (2025). Sprint running mechanics are associated with hamstring strain injury: A 6-month prospective cohort study of 126 elite male footballers. Br. J. Sports Med..

[9] Kalema R.N., Duhig S.J., Williams M.D., Donaldson A., Shield A.J. (2022). Sprinting technique and hamstring strain injuries: A concept mapping study. J. Sci. Med. Sport.

[10] Hagos A., Merchant A.A., Kayani B., Yasen A.T., Fares S., Haddad F.S. (2025). Risk factors and injury prevention strategies for hamstring injuries: A narrative review. EFORT Open Rev..

[11] Askling C., Tengvar M., Saartok T., Thorstensson A. (2000). Sports-related hamstring strains—Two cases with different etiologies and injury sites. Scand. J. Med. Sci. Sports.

[12] Opar D.A., Williams M.D., Shield A.J. (2012). Hamstring strain injuries: Factors that lead to injury and re-injury. Sports Med..

[13] Edouard P., Mendiguchia J., Guex K., Lahti J., Prince C., Samozino P. (2023). Sprinting: A key piece of the hamstring injury risk management puzzle. Br. J. Sports Med..

[14] Danielsson A., van Hooren B., Horvath C., Halvorsen K.A. (2020). The mechanism of hamstring injuries: A systematic review. Br. J. Sports Med..

[15] Tedeschi R., Giorgi F., Donati D. (2025). Sprint training for hamstring injury prevention: A scoping review. Appl. Sci..

[16] Ono T., Higashihara A., Shinohara J., Hirose N., Fukubayashi T. (2015). Estimation of tensile force in the hamstring muscles during overground sprinting. Int. J. Sports Med..

[17] Schache A.G., Dorn T.W., Blanch P.D., Brown N.A.T., Pandy M.G. (2012). Mechanics of the human hamstring muscles during sprinting. Med. Sci. Sports Exerc..

[18] Yu B., Queen R.M., Liu Y., Garrett W.E. (2017). Mechanism of hamstring muscle strain injury in sprinting. J. Sport Health Sci..

[19] Carmona G., Moreno-Simonet L., Cosio P.L., Astrella A., Fernández D., Padullés X., Cadefau J.A., Padullés J.M., Mendiguchia J. (2025). Acute changes in hamstring injury risk factors after a high-volume maximal sprinting session in soccer players. Sports Health.

[20] O’Sullivan K., Murray E., Sainsbury D. (2009). The effect of warm-up, static stretching, and dynamic stretching on hamstring flexibility in previously injured subjects. BMC Musculoskelet. Disord..

[21] Kenneally-Dabrowski C.J.B., Brown N.A.T., Lai A.K.M., Perriman D., Spratford W., Serpell B.G. (2019). Late swing or early stance? A narrative review of hamstring injury mechanisms during high-speed running. Scand. J. Med. Sci. Sports.

[22] Sople D., Wilcox R.B. (2024). Dynamic warm-ups play a pivotal role in athletic performance and injury prevention. Arthrosc. Sports Med. Rehabil..

[23] Evangelidis P.E., Shan X., Otsuka S., Yang C., Yamagishi T., Kawakami Y. (2023). Fatigue-induced changes in hamstrings’ active muscle stiffness: Effect of contraction type and implications for strain injuries. Eur. J. Appl. Physiol..

[24] Ruan M., Li L., Chen C., Wu X. (2018). Stretch could reduce hamstring injury risk during sprinting by right shifting the length-torque curve. J. Strength Cond. Res..

[25] Nagahara R., Mizutani M., Matsuo A., Kanehisa H., Fukunaga T. (2018). Association of step length and step frequency during sprinting with acceleration and maximum speed performance. J. Strength Cond. Res..

[26] Mendiguchia J., Alentorn-Geli E., Brughelli M. (2012). Hamstring strain injuries: Are we heading in the right direction?. Br. J. Sports Med..

[27] Higashihara A., Ono T., Kubota J., Okuwaki T., Fukubayashi T. (2010). Functional differences in the activity of the hamstring muscles with increasing running speed. J. Sports Sci..

[28] Chumanov E.S., Heiderscheit B.C., Thelen D.G. (2011). Hamstring musculotendon dynamics during stance and swing phases of high-speed running. Med. Sci. Sports Exerc..

[29] Ali S., Rafiq M.T., Ul-Hassan T., Sharif A. (2022). Role of warm-up exercise in preventing hamstring injury in sprinters. Rawal Med. J..

[30] Sugiura Y., Sakuma K., Sakuraba K., Sato Y. (2017). Prevention of hamstring injuries in collegiate sprinters. Orthop. J. Sports Med..

[31] Čoh M. (2013). Differences between the elite and subelite sprinters in kinematic and dynamic determinations of countermovement jump and drop jump. J. Strength Cond. Res..

[32] Bezodis N.E., Willwacher S., Salo A.I.T. (2019). The biomechanics of the track and field sprint start: A narrative review. Sports Med..

[33] Yu B., Queen R.M., Abbey A.N., Liu Y., Moorman C.T., Garrett W.E. (2008). Hamstring muscle kinematics and activation during overground sprinting. J. Biomech..

[34] Pandy M.G., Lai A.K.M., Schache A.G., Lin Y.C. (2021). How muscles maximize performance in accelerated sprinting. Scand. J. Med. Sci. Sports.

[35] Mendiguchia J., Alentorn-Geli E., Myer G.D. (2014). The role of hamstrings in the prevention of hamstring injuries. Sports Med..

[36] Hegyi A., Csala D., Péter A., Finni T., Cronin N.J. (2019). High-density electromyography activity in various hamstring exercises. Scand. J. Med. Sci. Sports.

[37] Cerone G.L., Nicola R., Caruso M., Rossanigo R., Cereatti A., Vieira T.M. (2023). Running speed changes the distribution of excitation within the biceps femoris muscle in 80 m sprints. Scand. J. Med. Sci. Sports.

[38] Li F., Guo C., Li H.S., Liu H., Sun P. (2023). A systematic review and network meta-analysis of the effects of different warm-up methods on the acute effects of lower limb explosive strength. BMC Sports Sci. Med. Rehabil..

[39] Bramah C., McLeod P., Wilson R. (2024). The sprint mechanics assessment score: A qualitative tool for assessing sprint running mechanics associated with lower limb injuries in male and female soccer players. J. Sports Sci..

[40] Gurchiek R.D., Teplin Z., Falisse A., Hicks J.L., Delp S.L. (2025). Hamstrings are stretched more and faster during accelerative running compared to speed-matched constant-speed running. Med. Sci. Sports Exerc..

